# In Memoriam. Dr. J. T. Webb

**Published:** 1889-09

**Authors:** F. S. White, W. P. Dumar, E. E. Smiley

**Affiliations:** President; Vice-President; Sec’y and Treas


### IN MEMORIAM.

At a called meeting of the Terrell Medical Society, held at the office of Dr. W. H. Monday, September 3, 1889, the following resolutions were passed:

Whereas, Death has removed from among us our friend and colleague, Jas. T. Webb, M. D.,

*Resolved*, That the members of the Terrell Medical Society, of which he was a member, and was associated with it from its very beginning, wish to express our deep sorrow for his providential removal by death.

*Resolved*, That in life and character of our departed associate in medicine, we recognize with pleasure his purity of character, and that his death will leave a void in our ranks much to be regretted.

*Resolved*, That in the death of J. T. Webb, M. D., we lament the loss of one distinguished for his amiability to his family and fidelity for M. E. Church, of which he was an indefatigable worker.

*Resolved*, That in the discharge of his professional duties he was conscientious, which endeared him to his fellow-men; he will long be remembered with deep affection, by rich and poor alike.

*Resolved*, That a copy of these resolutions be published in DANIEL'S TEXAS MEDICAL JOURNAL, and in the Terrell Times.

F. S. WHITE, President,
W. P. DUMAR, Vice-President.
E. E. SMILEY, Sec'y and Treas.

